# Traité des Nerfs et de leurs Maladies

**Published:** 1781-03

**Authors:** 


II. *Traité des Nerfs et de leurs Maladies. Par* M. Tiffot, M. D. &c. (Continued from page 108.)

OUR author confiders the catamenia as having confiderable influence on the nerves. The ill effects that fometimes take place place about the time of their ceſſation, he attributes to negligence or miſmanagement rather than to natural cauſes. He quotes almoſt the whole of what the late Dr. Fothergill has ſaid on this ſubject in the *Med. Obſ. & Inq.* M. Tiſſot remarks, that women who had been hyſterical till that period were then no longer irritable; he has ſeen others able to lay aſide ſpectacles, which before they had been obliged to uſe for ten years; and in one patient who had long been ſubject to a ſpaſmodic affection of the œſophagus, the complaint diſappeared after the ceſſation of the menſes. He ſpeaks of eryſipelas of the face as the moſt frequent complaint at this period.

Hippocrates long ago obſerved, that ſpaſm may be occaſioned by repletion or evacuation. Our author, therefore, conſiders the effects of plethora and hæmorrhage as liable to produce nervous affections. The latter ſeems to be the moſt frequent ſource of theſe complaints, by producing atony, and of courſe morbid irritability. He has ſeen a young woman, on the ſuppreſſion of a naſal hæmorrhage, to which ſhe frequently had been ſubject, affected with ſtrong convulſions, that gave way to repeated venæſections. He obſerves, that epilepſy often ariſes arifes from plethora, and quotes a cafe from Boerhaave of a man who by drinking Burgundy wine to excefs, was feized with a general fpafm of his whole body, but who recovered after lofing two pounds of blood.

M. Tiffot next treats of the effects of pregnancy, parturition, fuckling, and fluor albus on the nervous fyftem; and prefents us with a variety of inftances in which each of thefe caufes have proved the fource of nervous complaints.

He next confiders pain as a ftimulus, and of courfe as a caufe of nervous affection. He diftinguifhes ftimuli into, 1. Acrid humours, and, 2. Mechanical ftimuli. Under the firft head he quotes inftances of nervous difeafes brought on or relieved by the repulfion or return of cutaneous eruptions or gout; and fpeaks of the convulfions that have followed an abforption of cancerous virus, and even of ftagnant milk. He likewife notices the effects of acid in the primæ viæ. Under the head of mechanical ftimuli, he enumerates worms, flatus, biliary concretions, calculus, dentition, and bony excrefcences, or tumours irritating the brain or nerves.

Speaking

Speaking of the morbid ſenſibility of certain parts, he makes mention of two ladies who cannot take the mildeſt purge without being troubled with head-ach; and of another who vomits up every thing but cheſnuts. He knows an eccleſiaſtic, who having at the age of 23 had a violent cholic after eating cucumbers, has from that time, now 18 years ſince, felt a painful ſenſation, and occaſionally ſpaſms, in the part where the cholic was ſeated.

M. Tiſſot obſerves, that next to the paſſions of the mind, violent remedies are the moſt liable to produce nervous affection; that for inſtance, a purgative or an emetic given in too ſtrong a doſe are true poiſons, and prove hurtful by their ſtimulus.

In the ninth chapter the author treats of the moral cauſes of nervous diſorders, ſuch as the effects of intenſe application of the mind, of the imagination and paſſions; after which he deſcribes the ſympathies, beginning with thoſe of the brain and the other parts of the head, and proceeding to thoſe of the different parts of the body. In ſpeaking of the ears, he takes notice of their conſent with the lungs. Etmuller had formerly remarked, that by touching the meatus auditorius with a probe, we might excite a dry cough; and our author had occafion to experience the truth of this obfervation in a deaf patient at Laufanne. In the *Ephem. Curios. natur.* we read of a woman troubled with an obftinate cough, which could not be cured till the acrimony of the ear-wax that occafioned it was corrected. Pechlin faw an officer who vomited whenever this meatus was irritated; and there are not wanting inftances of perfons in whom mufic excites vomiting, or who feel an inclination to make water whenever they hear particular founds. In order to elucidate the fubject of nervous fympathy, M. Tiffot has added a table of the principal anaftomofes of the nerves.

In the eleventh chapter the author treats of metaftafes, coction, and crifis in nervous diforders. The firft of thefe, he obferves, were well known to the ancients. Hippocrates has remarked, that blindnefs, pain of the hips or tefticles, and fwelling of the breafts, cure epilepfy; and that a dry cough is removed by a palfy of the right hand and the left leg. Hoffman faw a tertian terminate in hyfteria, and a quotidian leave behind it periodical fpafms of the larynx and pharynx. Torti himfelf had a periodical deafnefs after a fever of of the ſame kind. Several other inſtances are added in this way.

In the following chapter, M. Tiſſot deſcribes the pathognomonic ſymptoms of nervous diſorders, and likewiſe the prognoſtic, and method of treatment. Under this laſt head he inquires into the merits of the general remedies uſually employed, as bleeding, evacuations, tonics, preparations of iron, volatile, and other ſtimulating ſubſtances, ſedatives, acids, gums, flores arnicæ, flores cardamine, zinc, milk, whey, baths, warm mineral baths, loadſtone, electricity, and mercurial frictions. He obſerves, that Manget ſpeaks of an hyſterical woman who was blooded one hundred and ſeventy-ſix times in leſs than two years, and who loſt ſeven ounces each time; and that M. Pomme ſaw a patient who had been blooded three hundred times. In both theſe caſes the evacuation had been productive of irreparable miſchief.

The author remarks, that purgatives and emetics will be of uſe only in the ſmaller number of caſes, when the irritation is kept up by the contents of the primæ viæ, and that the moſt ſuitable purgatives will be ſuch as are the leaſt irritating. Caſtor oil, we are

 told, told, will often fucceed, when every other irritates. With regard to tonics, he obferves, that in general their effects are foon difplayed. If they do not relieve the patient in a few days, we may conclude that it is neceffary to vary the treatment. He cautions us againft the ufe of fteel when there is a flow fever, confiderable fulnefs in the veffels, or a redundancy of bile in the primæ viæ. Under the head of volatile and ftimulating remedies, he fpeaks of veficatories, but feems to have overlooked their antifpafmodic powers, confining himfelf wholly to their ftimulus. He has often experienced the good effects of opium in violent fpafmodic affection, when there was no inflammatory diathefis, or difpofition to plethora. Acids he has found ufeful, 1ft. In the flow fever that produces all the fymptoms of nervous fevers, and that often yields to no other remedy; 2dly. In removing the irritability brought on by coffee, or by animal and aromatic food; 3dly. When nervous diforders depend on irritation from bile; 4thly. Whenever there is thirft and a quick pulfe. He generally confines himfelf to the acid of vitriol, and to that of oranges and lemons.

The

The gums he conſiders as poſſeſſing tonic, ſtimulating, and ſedative properties, but ſuppoſes them to act chiefly by the two firſt. The moſt efficacious, we are told, is aſſafœtida, for the uſe of which we are probably indebted to the Arabians. When the ſtomach is too ſenſible, the gums prove too irritating.

M. Tiſſot has employed the flowers of arnica only three times in palſy. He thinks it a dangerous remedy, where there is extreme irritability. The flos cardamine he has never tried, any more than the flowers of zinc; ſo that he contents himſelf with repeating what has been ſaid of theſe remedies by Sir George Baker and Gaubius.

Our author examines the properties of different milks, and in general gives the preference to aſſes milk. He obſerves, that when a milk diet is indicated, no preparation is required. He conſiders whey as the mildeſt of diluents. When there is extreme ſenſibility in the nerves of the primæ viæ, we are adviſed to employ aſſes milk; but when the irritation depends on bile, and the patient complains of heat, thirſt, nauſea, and high-coloured urine, the preference is then to be given to whey.

M. Tiſſot

M. Tiſſot conſiders baths, and eſpecially warm baths, as the principal means of cure in caſes of morbid irritability. This remedy has become much more general ſince Hoffman's time, but no phyſician has extended its uſe ſo far as M. Pomme, who ſometimes continues his patient in the warm bath four-and-twenty hours. Our author remarks, that the nervous caſes in which very hot baths are indicated are extremely rare.

He treats of the effects of cold mineral ſprings, and gives the preference to the Geronſtere water, as containing a volatile principle combined with iron, which renders it more efficacious than any other of the ſprings at Spa. He has ſometimes found the Seltzer water too ſtimulating.

With regard to the efficacy of the loadſtone, M. Tiſſot has himſelf had no opportunity of trying its effects; and on the ſubject of electricity, he contents himſelf with quoting what he had already ſaid on this matter in his letter to Haller, adding a few new facts by way of note.

He next conſiders the effects of muſic; and obſerves, that if any one wonders that muſic was preſcribed to Ulyſſes to cure him of the bite bite of a ſerpent; it is becauſe he is ignorant that nothing retards the healing of wounds ſo much as melancholy. Our author ſpeaks of a woman advanced in years, one of his patients, who had an ulcer on the hip, which during two years reſiſted every remedy; ſhe had been uneaſy about her ſon who was abſent; at the end of that time the young man returned, and ſhe ſoon got well. M. Tiſſot remarks, that muſic, though it may not deſtroy the cauſe of pain, removes the ſenſation of it, and thus obviates the irritation it occaſions, and of courſe contributes to the cure. He thinks, that by palliating pain, and promoting perſpiration, it may have a good effect in ſciatica and gout. Duke Albert of Bavaria is ſaid to have mitigated the gout by means of ſoft muſic; and Geſner ſpeaks of an Italian, who after labouring under ſciatica for a year, being animated by muſic to dance, danced every day for a week, and then found himſelf cured. In this laſt caſe, exerciſe ſeems to have had the greateſt ſhare in the cure; but M. Tiſſot, aware of this objection, very properly remarks, that no man afflicted with ſciatica would think of dancing, were he not exhilarated by muſic. Marſhal Saxe remarked, that his troops were always the leaſt least fatigued when they marched to beat of drum. When Achilles was furious, Chiron appeased him by means of his guitar; and Clinias made use of the same kind of instrument to calm himself when he was irritated. Sir Thomas More had recourse to music to soften his wife's temper. But the most circumstantial and authentic instance, and one almost as ancient as the siege of Troy, adds our author, of a nervous disorder cured by music, is that of Saul cured by David's harp. Asclepiades considered this fine art as an essential remedy in phrenzy, and all disorders of the mind; and Aretæus has recommended it against a kind of religious melancholy. Ancient history furnishes us with another proof of the power of music in the case of Alexander, whom Timotheus could animate or calm at pleasure. Many other instances are given by our author in this way; after which he points out the excellent effects of friction with a flannel or flesh-brush, and then speaks of the means to be employed in metastases, observing, that when they seem to relieve the patient, we ought to be careful not to counteract nature. He next speaks of the means of preventing nervous disorders, and obferves, that this will be beft done by avoiding their caufes.

In the laft volume, the author treats of epilepfy. In this, as in the other parts of the work, we meet with a judicious compilation of every thing that has been written on the fubject, but as this account of the epilepfy was publifhed feparately fo long ago as the year 1770, we prefume that our readers are already well acquainted with it; we fhall therefore pafs it over, efpecially as it affords no new light on the nature or treatment of the difeafe in queftion.

